# Iatrogenic Parasitic Leiomyomas: A Late and Uncommon Complication After Laparoscopic Morcellation

**DOI:** 10.7759/cureus.24718

**Published:** 2022-05-04

**Authors:** Harrypal Panesar, Harjit S Dhaliwal

**Affiliations:** 1 Otolaryngology, University Hospitals of North Midlands NHS Trust, Stoke-on-Trent, GBR; 2 Department of Obstetrics and Gynaecology, Royal Bournemouth Hospital, Bournemouth, GBR

**Keywords:** recurrent leiomyomas, myomectomy, parasitic leiomyomas, laparoscopic morcellation, disseminated peritoneal leiomyomatosis

## Abstract

Parasitic leiomyoma (PL) is an extremely rare variant of uterine leiomyomas that occurs outside of the uterus and can often present like intra-abdominal tumors. The aim of this study is to report a case of PL and compare it with current literature.

We present a rare case of a 45-year-old female who presented with bloating and spasmodic abdominal cramps for a two-month duration. She had a previous laparoscopic myomectomy six years ago. Transvaginal ultrasound (TVUS) showed solid vascular masses in the pelvis, the largest being 6 cm. Computed tomography (CT) of the thorax, abdomen, and pelvis (CTTAP) revealed further peritoneal masses in the left paracolic gutter suggesting peritoneal distant metastasis. Laparoscopy was completed, and biopsy and histopathological examination confirmed the diagnosis of parasitic leiomyoma. The patient opted for a bilateral salpingo-oophorectomy (BSO) creating iatrogenic menopause. One-year follow-up CT showed a reduction in the size of fibroids.

PL can present with vague symptoms, typically nonspecific abdominal pain and cramping. It can often be confused with intra-abdominal tumors. It should be suspected in patients with previous uterine procedures. Histopathological examination is crucial for diagnostic and surgical management.

## Introduction

Parasitic leiomyomas (PL) are a rare manifestation of uterine leiomyomas. Classically, they are described as pathological entities that may occur spontaneously due to a pedunculated subserosal fibroid lying away from the uterus that develops a blood supply from adjacent structures [1]. Kelly and Cullen first described PL in 1909 [2], but only in recent years, with the emergence of laparoscopic surgery, has PL been described as having an iatrogenic origin, particularly following laparoscopic myomectomy with the use of a morcellator [3,4].

Patients with PL often present with vague symptoms of abdominal pain, distension, and a sensation of a mass in the abdomen or pelvis. Management of PL is usually with surgical resection, but candidates unsuitable for surgery can benefit from medical management in the form of ovarian suppression therapy.

The overall incidence of iatrogenic parasitic myomas after laparoscopic surgery with the use of morcellation is reported to be 0.12%-0.95%, while the reported incidence after laparoscopic myomectomy is 0.20%-1.25% [5]. The time interval between the initial surgery and diagnosis is reported to be between three and eight years [6].

In keeping with Surgical CAse REport (SCARE) guidelines [7], we present a case of PL in a 45-year-old female for whom the initial diagnosis at presentation, based on her symptoms, elevated tumor marker levels (CA125), and imaging results, was of a suspected primary ovarian malignancy.

## Case presentation

**Figure 1:**
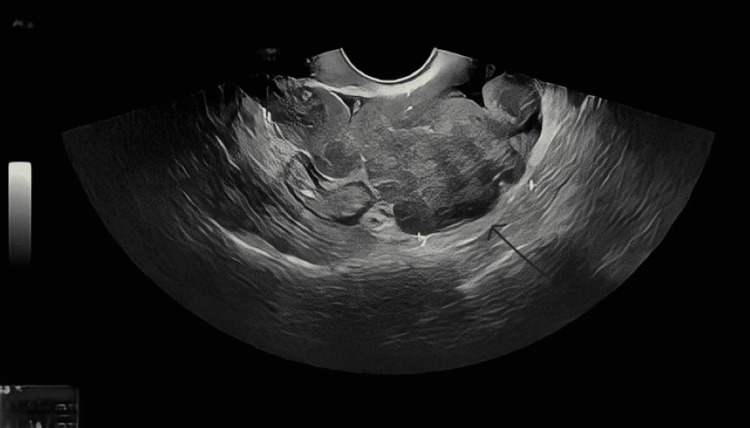
Heterogenous mass in the pouch of Douglas

**Figure 2:**
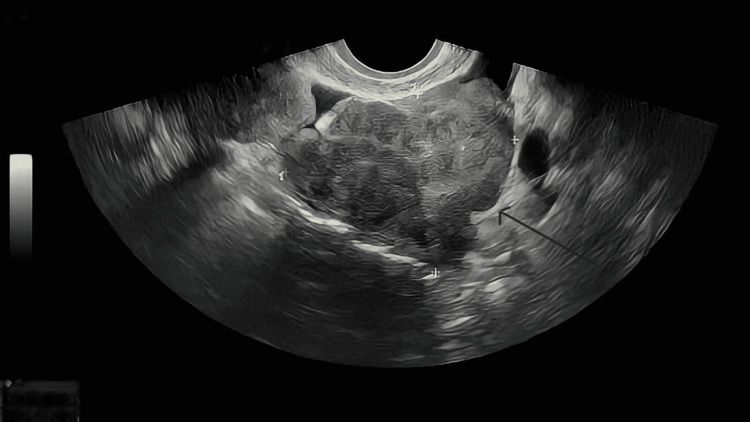
Heterogenous mass in the left adnexa

A 45-year-old nulliparous female was referred to the ambulatory assessment clinic with a two-month history of feeling bloated and experiencing spasmodic abdominal cramps and with a raised CA125 of 134 kU/L (normal range: 0-35 U/mL). Other tumor markers (CEA and CA19-9) were within normal limits. Her past medical history was unremarkable, apart from a laparoscopic myomectomy six years previously for a large, 10-cm posterior wall fibroid. Her social history revealed that she was a nonsmoker and nondrinker. In view of her symptoms and the elevated tumor marker, an urgent transvaginal ultrasound (TVUS) was requested, which suggested several solid vascular masses throughout the pelvis (Figures 1, 2), the largest of which was about 6 cm in diameter, with a moderate amount of pelvic free fluid.

Following the TVUS, she underwent a computed tomography (CT) of the thorax, abdomen, and pelvis (CTTAP), which revealed multiple enhancing bilateral adnexal lesions and further lesions in the left paracolic gutter, all of which were suggestive of peritoneal distal metastasis. There were also two indeterminate 2-mm pulmonary nodules in the periphery of the right upper lobe.

Due to her history of previous myomectomy, her histopathology results, and the unusual CT appearance, PL or even disseminated peritoneal leiomyomatosis were suggested, with the further possibility of a benign metastasizing leiomyoma as an incidental lung nodule. Further differentials included metastatic ovarian carcinoma.

**Figure 3:**
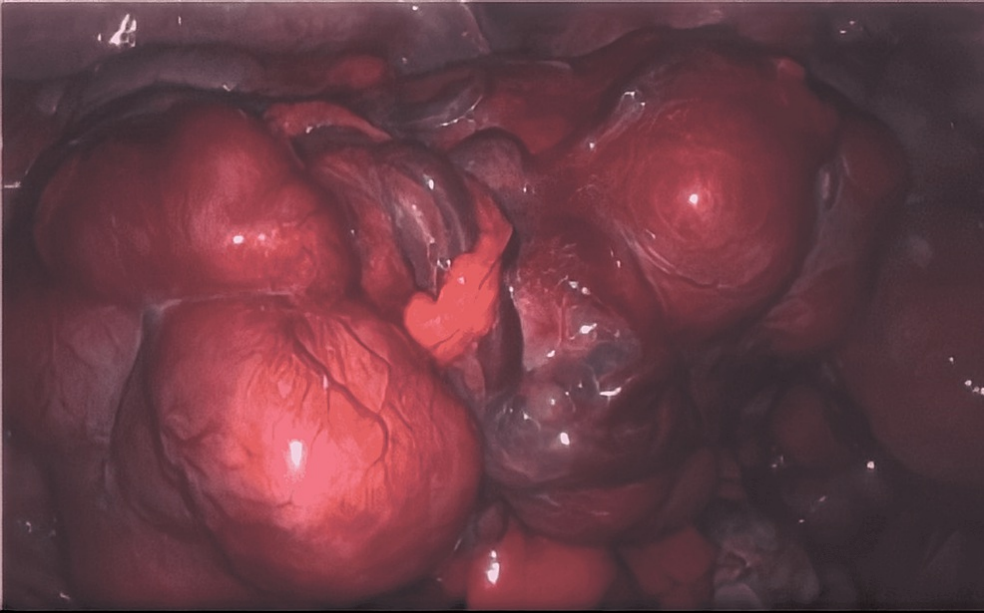
Intraoperative laparoscopy image depicting multiple solid masses in the pouch of Douglas

A diagnostic laparoscopy was performed, and a biopsy confirmed the presence of benign leiomyomas (Figure 3). The omentum was clear of any smooth muscle nodules, and cytology for the ascitic fluid was negative for malignant cells. There were multiple large solid masses within the pouch of Douglas, in the left ovary/adnexa, within the uterovesical fold of the peritoneum, on the colonic serosa of the sigmoid colon, and attached to the peritoneum in the left paracolic gutter. A small nodule was also found on the anterior abdominal wall at the previous laparoscopic suprapubic entry point.

The patient was extensively counseled regarding ovarian suppression either by medical therapy or by a surgical option that would entail a bilateral salpingo-oophorectomy (BSO), which would create iatrogenic menopause to bring about a secondary reduction in the fibroids, especially those involving the sigmoid colon. She opted for the latter. A follow-up repeat of the CT of the thorax three months after surgery suggested no further changes to the lung nodules. A further CT scan one-year post-surgery showed an overall reduction in the size of the fibroids and no lung nodules.

## Discussion

Iatrogenic PL is a rare condition resulting from the regrowth of fibroid fragments that have been displaced and left within the peritoneal cavity after laparoscopic morcellation during a myomectomy or hysterectomy [8,9].

The most widely accepted theory of the pathogenesis of PLs states that they arise from previous subserosal pedunculated leiomyomas that undergo torsion around their peduncle and subsequently lose their attachment to the uterus. Revascularization of separated uterine leiomyomas is achieved via mesenteric and omental vessels. Other theories include peritoneal metaplasia, which describes the development of myomas in unexpected areas of the abdomen [10]. However, with the widespread use of laparoscopy in gynecological procedures, the current most recognized theory of their origin is tissue translocation and implantation in the abdominal and pelvic regions attributed to the use of morcellators.

The development of uterine leiomyomas is known to be influenced by gonadal steroid hormones, specifically the duration of exposure [11]. In a similar fashion, it has been proposed that prolonged exposure to steroid hormones can be a risk factor for the development of PL. Takeda et al. found remarkable similarities in the histological appearance of PL in patients who had undergone laparoscopic myomectomy with morcellation six years previously and also reported that PLs experience rapid growth during pregnancy, supporting the role of sex hormones in their growth [12].

PL is most commonly located in areas with a good blood supply, which include the abdominal peritoneum, pouch of Douglas, sigmoid colon, broad ligament of the uterus, greater omentum, and laparoscopic entry points [13]. Other locations can be under the diaphragm [14] and within the lung, where it is also described as benign metastasizing leiomyoma [15]. The radiological findings of pulmonary nodules in benign metastasizing leiomyomas can vary from an appearance of small solitary nodules to multiple lesions mimicking metastatic pulmonary disease from malignant tumors. The appearance of such lesions has also been reported to have a miliary pattern [16] and to mimic interstitial lung disease [17]. In our report, two indeterminate sub-centimeter lung nodules were noted on CT imaging, consistent with descriptions in reports by Wolff et al. [18]. Although not confirmed by histopathological examination, these lesions are thought to have been a metastasizing leiomyoma from the extensive disease within the uterine structures, and this theory is supported by the resolution of the nodules on follow-up imaging after the surgical iatrogenic menopause.

PL can be asymptomatic, but common symptoms are abdominal pain, abdominal pressure or mass, vaginal bleeding, abdominal distension, dyspareunia, urinary frequency, and constipation [10]. The diagnosis is often incidental and is found through radiological examination or during laparoscopy conducted for another reason. PL poses diagnostic challenges because intrabdominal leiomyomas can mimic malignancy due to their unusual locations, growth patterns, and sizes.

The management of PL is usually surgical excision, but sometimes, lesions can adhere to abdominal viscera, and excision can thus be risky [19]. In such cases, medical management or other safer forms of surgical management can bring a reduction in the size of the parasitic myomas. There is no consensus on the preferred method for treating and preventing the recurrence of PL, and BSO might not be indicated in all patients. The use of aromatase inhibitors and selective progesterone receptor modulators (SPRMs) has a significant effect on the reduction of uterine myoma implantations in comparison to gonadotrophin-releasing hormone (GnRH) agonists and selective estrogen receptor modulators (SERMs) [20].

## Conclusions

PL is a common benign tumor that can present as an intra-abdominal malignancy. It is important to consider PL in differential diagnosis, especially in patients with a history of laparoscopic morcellation or hysterectomy. Patients should be counseled about this serious late complication, the effects it might have in the future, and the risks of extensive and complicated debulking surgery, such as omentectomy and bowel resection. Surgical intervention proved effective in this case in reducing the tumors, but medical treatments are also available for nonsurgical candidates.
